# P-2251. The Infectious Complications After Belatacept-Use in Kidney and/or Pancreas Transplant Recipients

**DOI:** 10.1093/ofid/ofae631.2404

**Published:** 2025-01-29

**Authors:** Abdinoor Abdi, Wyatt Tarter, Hareesh V Singam, Adam Bregman, Karam Obeid

**Affiliations:** University of Minnesota, Minneapolis, Minnesota; University of Minnesota, Minneapolis, Minnesota; University of South Florida, Tampa, Florida; University of Minnesota, Minneapolis, Minnesota; University of Minnesota, Minneapolis, Minnesota

## Abstract

**Background:**

Conversion therapy substitutes belatacept (bela) for calcineurin inhibitors (CNI) after kidney and/or pancreas transplant (KT and/or PT). Investigations have yielded conflicting findings regarding the incidence of infections associated with bela- compared to CNI-use. This study explores the correlation between bela-use following KT and/or PT and the occurrence of fungal, CMV, EBV, and BKV infections, alongside mortality rates.

**Methods:**

We included patients with a primary KT and/or PT between 1/1/2018 and 12/31/2022 and allowed for a 6-month follow up until 6/30/2023. We compared infectious outcomes in those who received bela at any time after transplant to those who did not. Cox proportional hazards models were used with bela as a time-varying covariate, and they were adjusted for gender, age at transplant, donor type, primary disease leading to transplant, CMV serostatus, and organ transplant type. Prentice-Williams-Peterson Cox models were used to examine the outcomes related to the recurrence of the viremia.

**Results:**

We identified 558 unique KT and/or PT recipients, of whom 30 received bela after transplant as a conversion therapy. The mean (±SD) duration of bela was 19 months (±18) and 22 doses (±19). After bela-use, the adjusted hazard ratio [HR (95% CI)] for the first CMV, EBV, and BKV viremia in bela patients was 2.48 (1.08, 5.73; p< 0.05), 4.30 (2.22, 8.34; p< 0.05) and 1.01 (0.37, 2.75) times that of control patients, respectively. The HR for recurring CMV, EBV, BKV viremia was 1.54 (0.86-2.77), 2.71 (1.23, 5.97; p< 0.05), and 0.45 (0.16, 1.21), respectively. The HR for fungal infections was 2.29 (0.52, 10.1) for bela patients. The HR for mortality was 4.14 (1.95, 8.79; p< 0.05). An increase of age by a year was associated with higher mortality [HR 1.06 (1.03, 1.08; p< 0.05)], and CMV [1.02 (1.00-1.03; p< 0.05)], EBV [1.02 (1.01-1.03; p< 0.05)], and BKV [1.02 (1.01-1.03; p< 0.05)] viremia.

**Conclusion:**

In this population of KT and/or PT recipients, bela-use as a conversion therapy is associated with a higher risk of mortality, CMV and EBV viremia, as well as recurrent EBV viremic episodes. Physicians should be especially vigilant when using bela for the older population.

**Disclosures:**

All Authors: No reported disclosures

